# Value-creation in the health data domain: a typology of what health data help us do

**DOI:** 10.1057/s41292-022-00276-6

**Published:** 2022-04-12

**Authors:** Amelia Fiske, Alexander Degelsegger-Márquez, Brigitte Marsteurer, Barbara Prainsack

**Affiliations:** 1 Institute of History and Ethics in Medicine, School of Medicine, Technical University of Munich, Munich, Germany; 2 Austrian National Public Health Institute (GÖG), Vienna, Austria; 3 Department of Political Science, University of Vienna, Vienna, Austria

**Keywords:** Health data, Value, Covid-19, Regulation, Data type

## Abstract

It has become a trope to speak of the increasing value of health data in our societies. Such rhetoric is highly performative: it creates expectations, channels and justifies investments in data technologies and infrastructures, and portrays deliberations on political and legal issues as obstacles to the flow of data. Yet, important epistemic and political questions remain unexamined, such as how the value of data is created, what data journeys are envisioned by policies and regulation, and for whom data types are (intended to be) valuable. Drawing on two empirical cases, (a) interviews with physicians on the topic of digital selfcare, and (b) expectations of stakeholders on the use of Real-World Data in clinical trials, as well as existing literature, we propose a typology of what health data help us to do. This typology is intended to foster reflection about the different roles and values that data use unfolds. We conclude by discussing how regulation can better accommodate practices of valuation in the health data domain, with a particular focus on identifying regulatory challenges and opportunities for EU-level policy makers, and how Covid-19 has shed light on new aspects of each case.

## Introduction

The Covid-19 crisis has turned health data into a household term. While debates about data quality, interoperability, and access may have previously been restricted to largely expert circles, with the pandemic these questions became the topic of prime-time news and trends on social media. For example, Tomas Pueyo’s data analytics about the spread of Covid-19 infections piqued the interest of millions in epidemiological research and assessments (Pueyo 2020). The reading of infection data dashboards and case-load updates became part of many people’s daily media consumption. Analyses of geocoded infection and mobility data were debated in relation to questions of whether ‘hard lockdowns work’ (Heiler et al. 2020). Models of how airborne particles travel illustrated potential exposure for daily activities such as going to the grocery store (Guarino and Achenbach 2020). While there were heated debates on- and off-line about how to interpret infection data and about what data are missing, the value of health data never seemed to come into question.

What is health data? How does it become valuable? The EU’s General Data Protection Regulation (GDPR) defines ‘data concerning health’ as “any personal data related to the physical or mental health of a natural person, including the provision of health care services, which reveal information about his or her health status” (General Data Protection Regulation (GDPR) 2016, p. Recital 35). This seems to limit the remit of health data to *personal* data, namely data that is related to an identified or identifiable natural person—excluding anonymous and anonymised data that cannot (or can no longer) be linked to any specific individual (meaning that pseudonymised data are considered personal data and thus within the remit of the GDPR). As Purtova (2018) and others have argued, however, because personal data is not only data that links to an already identified individual person, but also data that could lead to the identification of a specific individual, the remit of personal data is very broad—and extends to IP addresses, mobility data, and consumer data. In addition, the GDPR’s formulation that health data is personal data ‘related to’ the health of people also in the sense that it “reveal[s] information about her health status” (GDPR), this makes the remit of health data even wider: It is possible to link almost every dataset with others and discover associations from which health-related characteristics can be inferred—even if the original datasets are not immediately health-relevant (Prainsack, 2017; Prainsack and Van Hoyweghen, 2020). For example, if seemingly innocuous characteristics such as the purchase of certain types of consumer goods online, or the watching of daytime television, are associated with health-relevant outcomes, then the initial data on online purchases or the consumption of TV streaming services has become health-relevant as well (Duhigg, 2012). In this sense, all types of data can yield information that is relevant to health—a fact that regulation does not adequately accommodate.

**Fig. 1:**
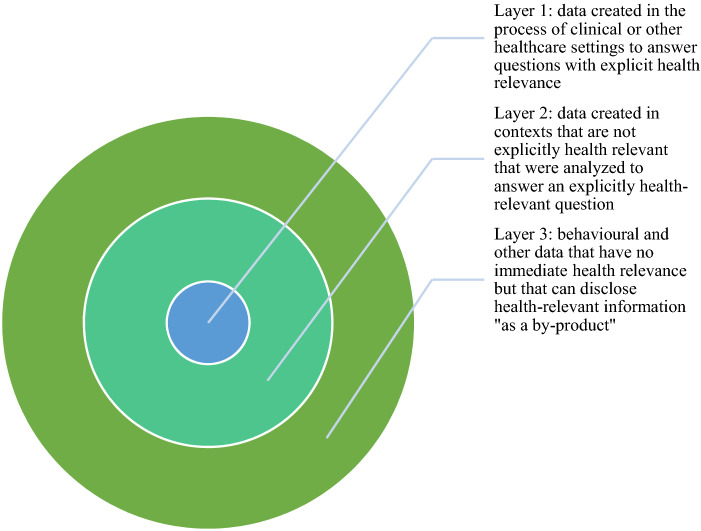
Layers of health data. (Color figure online)

This does not mean, of course, that all data types are health-relevant in the same manner. It is helpful to distinguish at least three layers of health data (Fig. 1). The first layer includes data that was collected and analysed in an explicitly health-relevant context, such as health records, lab tests, results from clinical genetics examinations. The second layer consists of data that was collected in other, not explicitly health-relevant contexts but that is, or can be relevant, to answer a health-relevant question—such as a person’s activity tracker data or consumption habits. The third layer is data that may seemingly have nothing to do with health, but that could disclose health relevant information as a byproduct, or if linked with other data—such as holiday pictures, credit card purchases or social media postings (Weber et al. 2014). The third layer also includes data relating to the ways that individual health is co-determined by socio-economic factors, education, behavior, environment, location, and more.

In exploring value-creation in the health data domain, we begin our reflections on the notion of value. In a market setting, products and services are valued by consumers and producers and prices are attached accordingly. Of course, not everything that has value has a price (our personal health, for instance), and things with the same price tag can have very different values for different people (the price of a heart implant, for instance). Value is not intrinsic to a specific product or service, but depends on context and relationships (e.g. between provider and user). This turns our attention from value to valuation as a social process that each of us is deeply involved with in a variety of settings. Different subfields of sociology are engaged with studying the basic social process of valuation and evaluation—from cultural and economic sociology, to inequality studies and sociology of science (see also Lamont (2012). Building on Lamont, Helgesson and Muniesa outlined a research programme for ‘valuation studies’ that understand value as a social construct—not in the sense that value would only be the outcome of shared belief, but of organised social work aimed at making things valuable (2013, p. 6). The result of this organised social work in some cases can be the objectification of a given value, one that is materially consequential albeit historically contingent (Mitchell and Waldby 2010).

We understand the perceived and enacted value of data as an example of an outcome of materially consequential, but historically contingent, valuation practices. Whether at the policy level, in public administration, in many realms of economic activity, or in scientific activity, different actors have a range of expectations about how value is drawn from data. This ranges from the ‘data as oil’ metaphor to more sector-specific discussions in manufacturing or health. According to Wilson and colleagues, one important element in determining the value of a specific dataset is the purpose for which it can and will be used. The value of data is also always influenced by relational factors (Wilson et al. 2020): not only because the value of a dataset can be influenced by who curated or collected it (e.g., because the data curator is known to be particularly rigorous, or because data from particular data sources or subjects are difficult to obtain), but also because different actors value the same dataset differently. Sometimes the same actors attach different types of value to the same dataset, e.g. financial value and personal value. A patient might value his or her health monitoring data as a reassurance of a current health status. He or she might also value it financially if offered an opportunity to do so. It is also for this reason that value is not completely separable from the process of valuation: Rather than being the *result* of valuation, different types of value serve as reference points for the practice of making something valuable.

While data are typically considered nonrival in that one person’s use of the data does not detract from the use of the data by others, and durable in the sense that they do not depreciate and are not destroyed by consumption, unlike tissue or DNA samples, the same does not necessarily apply to its value: “Data that provide a competitive advantage will lose much of their value [for a given actor] if they become common knowledge” (Wilson et al. 2020, p. 2). In the online space, the data that individuals provide through their social media activity can become the main assets of companies’ data infrastructures (Tempini 2017). In politics and public administration, certain actors can derive value from the mere (and repeated) call for more data (Hoeyer 2019). In the realm of science, powerful analytical tools might indeed be available to create value (financial, epistemic, etc.) out of large amounts of data available to academic actors—the problem is moving the data around. This requires solutions to a number of social, ethical and semantic challenges (Leonelli 2019), and these solutions are not always available to all. Work is required to draw value from data, and the capacity to carry out this work (curate large amounts of data, hold data in storage infrastructure, sell data in the right form at the right time, etc.) is unevenly spread.

Also in the health domain, the value of data is historically contingent, purpose-specific, and relational. Organised work goes into making health data valuable. Traditionally, this is linked to the ‘primary use’ context: health data are collected in healthcare settings for the benefit of the patient. Primary use data infrastructures are developed with the aim of improving health outcomes. Prior to healthcare practice, other major data infrastructures are built for clinical research on diagnostics and treatment options. Health data are produced and analysed in highly controlled experimental environments, with data curated in dedicated clinical study software.

The same data that for some help to develop treatment options or monitor health status (e.g. through smart patient devices) are a source of revenue for others (e.g. the pharmaceutical industry and device manufacturers). For yet another group, these data gain value in a secondary use context, i.e. where they are used outside the initial purpose of data collection, such as when public health and pandemics management leverage health data initially collected for other purposes (Budd et al. 2020); clinical studies draw from routine data, or registry data or patient-collected data are used to complement data collection in traditional randomised controlled trials (Bolislis et al. 2020, p. 927). Accordingly, the value of health data might be measured, for example, in terms of clinical outcomes, or on a scale of individual wellbeing and empowerment, in monetary units, or in quality-adjusted life years (e.g. when assessing the population-level effects of data-driven digital health interventions).

Some actors in the health data space have sought to establish themselves as platforms, with business models built around the idea of creating different types of value with the same kind of data. Tempini (2015) investigated the example of the company PatientsLikeMe, which allows patients to share self-reported medical data while also offering the data to medical research. In order to enable both communities to benefit from the data (and to draw financial value from it for the company’s purposes), the conflicting demands of sufficient patient engagement (necessary to get data at scale) and sufficient semantic context (to achieve a suitable degree of specificity in the data) need to be balanced. Other authors have focused on various forms of ‘data work’ necessary to produce and, ultimately, to draw value from data in healthcare and related fields (Fiske et al., 2019a, 2019b) Medical scribes, a profession dedicated to clinical documentation alongside physicians, collect data that is imported into electronic health records (Bossen et al. 2019); data curators package ‘small facts’, physical traces left by experimental apparatuses, for data journeys (Leonelli 2010); researchers invest in long-term care relationships with their data (Pinel et al. 2020). The various efforts different actors invest in health data valuation has thus already been the subject of significant attention.

What is missing, to the best of our knowledge, is a systematic overview of the roles data can assume for actors in the health space, or, in other words, what it is that health data helps actors to do. Such a systematic overview is also necessary if we seek to reform regulation to better fit data use in the digital era. In reflecting on the Covid-19 pandemic, and increasing calls for the need for ‘more’ data, or the inherent value of data in devising appropriate public health responses, we argue it is productive to shift the focus away from generalised notions of data that are considered sensitive or not, or data that can ‘facilitate research’ or ‘improve healthcare’ to instead consider data in terms of roles and uses, within a suite of relationships and practices that can, in turn, affect value. We move towards this shift in approach through the creation of a typology of the roles of health data, which we have inductively derived from two empirical case studies conducted by the authors, and is informed by the above literature on value and health data. Our intention is to focus on what is actually being done by and with health data: the roles it plays, the forms of value it creates, and for whom. This focus has specific relevance for examining the role of health data in regulation, in unfolding crises such as the pandemic, and can be expanded for considering the shifting uses and applications of health data in society.

## Methods

This paper draws upon two empirical case studies conducted by the authors. The first one revolved around reflections on digital self-care (DSC) by physicians in Northern Germany, and the other on expectations of the use of Real-World Data (RWD) in clinical trials by those working for institutions that run or participate in these trials. Each case provides important insights for thinking about the roles that data play in the health domain (both in research and practice), and the kinds of value (Burton et al. 2021) they are involved in creating. We used our analyses of these cases to explore the different ways through which health data becomes valuable. On the basis of an inductive process of collaborative coding, reflection, and writing, we distilled our findings from the two cases in a typology of different functions that data has, and the types of values that emerge from them.

### Data collection for the digital self-care study

In this study, two of the authors (AF and BP) explored physician perspectives on how digital self-care (DSC) practices are encountered, understood, and incorporated (or not) into the healthcare system. We understand digital self-care to encompass practices undertaken by patients that include both novel and more traditional forms of participation. Digital self-care comprises both digital practices that used to be analog, such as the writing of mood diaries or tracking one’s sleep, as well as practices that have only become possible only through novel forms of datafication, such as activity tracking or forms of diagnostic testing conducted at home) (Fiske et al., 2020). Ethics approval was received on 16 October 2017, by the Christian-Albrechts-Universität Kiel Research Ethics Commission (D 548/17) .

Participants were recruited by email, with an explanation of the project and invitation to participate. Participants were selected largely through snowball sampling; several respondents recommended other physicians to be invited for participation. All participants were physicians or professors working in areas relating to digitisation and self-care in medicine. Attention was paid to the recruitment of respondents across a range of medical specialties, including: geriatrics, neurogeriatrics, diabetology, pediatric diabetology, obstetrics, clinical genetics, dermatology, neurology, radiology, psychiatry, neuropediatrics, gastroenterology, emergency medicine, internal medicine, and general practice; some respondents had additional training in medical business management and telemedicine. Participants had a varying range of professional experience from two to several decades. In total, six women and nine men participated.

The interviews were carried out in the English language and lasted between 30 and 90 min. They followed a semi-structured format. All informants gave consent to be audio recorded, and were given the opportunity to review the transcript of their interview. All respondents spoke German as a first language but had a high degree of English-language fluency. All interview participants were asked the same set of questions following a two-part interview format. The first part elicited respondent understandings of self-care with open-ended questions. The second part of the interview guide consisted of two hypothetical scenarios about the use of direct-to-consumer diagnostic testing and self-tracking devices, which were chosen to capture two distinct areas of possibility and potential concern within the domain of DSC practices. Interviewees were asked to respond to these scenarios, and were asked questions about potential concerns, benefits, and applications of the examples. Resulting transcripts were pseudonymised, and potentially revealing information was removed.

### Data collection for the Real-World Data study

This project focused on stakeholder expectations regarding the benefits of using Real-World Data (RWD) in clinical trials. RWD are “data relating to patient health status and/or the delivery of health care routinely collected from a variety of sources” (FDA 2018, p. 4), i.e. they are collected outside the controlled environment of clinical trials. Key sources are claims and billing databases, patient and product registries, and all types of patient-generated data. Real-World Evidence (RWE) is clinical evidence that is derived from the analysis of RWD. The authors (ADM and BM, both of whom work at at The Austrian National Public Health Institute (GÖG), combined a desk-based literature study with a set of exploratory interviews to substantiate the theoretical structure for the study. Interviews included individuals who work in the research-based pharmaceutical industry and at university hospitals where clinical research takes place, and for regulatory bodies. One interview included a health start-up whose business model is based on RWD and RWE.

Participants were selected because of their expertise or their role within their institutions and were recruited by e-mail and provided with information about the research project and a consent form. Five interviews were conducted in German and one in English via videoconferencing tools, depending on preferences of the interviewee, and lasted between 30a and 60 min. Findings of the interviews conducted in German were transcribed and then translated into English. All participants gave their consent to the audio recording of the interview for transcription. Six men and no women participated.

In the first part of the interview, participants were asked to share their views about the role of data in clinical research, and in particular on data collection. The second part of the interview focused on reflections and visions about RWD and RWE. The interview guide was adapted accordingly based on the role and expertise of the interviewee, e.g. if a respondent was already explicitly involved with RWD and RWE in an institutional role. Transcripts were pseudonymised, and potentially revealing information was removed.

### Data analysis and conceptualisation

Specifically, we proceeded in the following manner: Each team of two authors (DSC study: AF and BP; RWD study: ADM & BM) thematically coded the interviews of their respective studies. Initially, transcripts were coded independently by each author. Data were subsequently evaluated in line with the principles of qualitative content analysis and constructivist Grounded Theory (Charmaz, 2014), in order to identify novel themes from the data. Following this, each author discussed coding results, and refined sub-themes to ensure a high degree of inter-coder reliability, with the aim of identifying different roles that data play in health research and medical practice, and extrapolating different types of value that they yield. All four authors met repeatedly to generate and consolidate the roles that they had drawn from their data to create a typology. Specific attention was paid to the use of health data during the Covid-19 pandemic, between the period of January 2020–April 2021. This included reflections drawn from the involvement of two of the authors (BP and AF) in a longitudinal, qualitative 9-country study on the pandemic (“Solidarity in times of a pandemic, SolPan”), as well as literature review. Given that the Covid-19 pandemic is ongoing, and the use of health data will no doubt continue change over time both in relation to the pandemic and more generally, we expect that the roles of health data will continue to evolve and expand.

The typology was empirically derived from DSC and RWD cases. The case studies, combined with our reflection and review of literature relating to the uses of health data during the Covid-19 pandemic, helped us to produce the first iteration of the typology. The resulting typology of data roles was then sent to health data researchers for comment; their comments were used to refine the typology further until it was precise enough to capture the majority of roles of data and types of value produced. Thanks to this input and rounds of subsequent revisions, the typology has grown from its initial version. As the typology grew, we deliberately elected to not only use examples from the case studies, but to broaden the remit and relevance of the typology by bringing in other examples.

The typology is meant to be an opening towards considering the range of functions of health data. The categories in the typology are not meant to be exclusive or exhaustive: There are many examples which speak to multiple categories at once, and the typology is intended to be read, used, and added to with this kind of fluidity in mind.

## Results: typology of roles of how health data become valuable

Drawing on the DSC and RWD study, combined with literature review of data valuation practices and reflection on the use of health data during the Covid-19 pandemic, we have created a typology (Table 1) exploring how data can become valuable in the health domain. The typology identifies the roles of digital data in the health domain, asking: *What can data (help actors to) do? What value does the data create? What practices affect the value of the data?* Below we elaborate on each of the roles of digital data that we have identified, with the caveat that the typology does not aim to be exhaustive nor are the categories intended to be exclusive. Points of overlap and convergence are seen across the different roles. We deliberately opted for a ‘flat’ typology in the sense that it is not informed by any hierarchy. We did so because we understand value and valuation as entities that mutually shape each other and are inseparable. While one practice may comprise only one type of valuation—and generate only one type of value, such as financial value, more often than not, different kinds of value and valuation shade into one another in practice.

**Table 1:** How data can become valuable in the health domain

	Roles of digital data in the health domain: What can data (help actors to) do?	What value do the data create?
A	Observing and interpreting	Enables sense- and meaning-making through empirical observation, e.g. to reconstruct actors’ interpretation of a situation
B	Quantifying and classifying	Connects two entities (e.g. symptoms to a diagnostic group), helps with clustering, sorting, and ordering
C	Generating and testing hypotheses	Creates evidence out of data to develop or test hypotheses
D	Enabling automation	Enables processes that were done by humans to be done by machines
E	Changing or stabilising hierarchies and power positions	Changes the distribution of power, agency, and resources between actors
F	Changing or stabilising practices and procedures	Provides new insights, or incentivising certain practices (e.g., activity trackers)
G	Proxying	Creates a ‘digital twin,’ or inputs data where they are missing, enabling experiments or interventions that would otherwise not be possible
H	Predicting	Models possible situations and outcomes
I	Increasing operational efficiency	Leads to efficiency gains in care delivery (data-enabled self-monitoring) or clinical research (for instance, more precise recruitment and/or site selection in clinical trials)
J	Generating financial profits	Accrues financial profits for different actors (e.g. capital investments)
K	Creating value through expectation	Generates value through the expectation of future value
L	Stimulating debate and deliberation	Illuminates spaces of theoretical and applied differences, conflicts, or questions

### A: Observing and interpreting

Data gained from the observation of patients has long had important clinical value for making health care decisions. These data can be interpreted in relation to changes over time, correlations or in relation to other data-based criteria. Further, partly due to the greater availability of digital tools and molecular technologies, ever wider aspects of people’s bodies and behaviors become observable and are being datafied (i.e. captured in digital data). This process yields larger amounts of health data that clinicians or researchers can interpret. Patients themselves also use the data gained from empirical observation for self-oriented sense-making affecting their interpretation of a situation. This happens, for example, when a pregnant woman entering fetal movements into an app considers the app part of her sensory instruments.

Bodily observations, whether collected through analog observations or digital tools, can also influence personal or clinical decisions, and contribute to meaning-making around one’s health and body. As Weiner et al. (2020) have shown, close attention to how data is, or is not recorded, can provide insight into how self-monitoring data flows (or does not flow) to big data sets. For example, a patient trying to eat a specific diet may remember what she ate in the last two or three days, but certainly not a few months ago; if she did not keep a diet journal there would be no way to access this information. The same patient using a diet app where she uploads pictures of everything she eats and enters the approximate size (or calorie count) creates a dataset that is available for interpretation for a long time, both for her personal use and, theoretically, for others as well. The resulting data can be useful on multiple levels, for the person herself, for their care providers, or within the context of a larger data set for a researcher or a company interested in deriving user insights. Physicians in the DSC study echoed this idea, noting that self-tracking devices could encourage self-observation, which may be personally valuable for patients in managing their health. We also observe this trend of datafication facilitating observation and interpretation in the context of Covid-19, e.g. with regard to self-testing: a new type of health data becomes individually and socially meaningful when the SARS-CoV-2 infection status is relevant knowledge for participation in public life.

### B: Quantifying and classifying

Data can connect two or more entities (e.g. symptoms to a diagnostic group), and help with clustering, sorting, and ordering. Medical research and practice have always been about classifying. In its most basic form, different types of health data are used to classify individuals as sick or healthy. Patients are routinely classified, whether on the basis of their disease (‘diabetic’), their probability for developing a particular disease (‘at risk’), or on the basis of specific health traits (‘obese’). Patients are also clustered by type of disease or risk factor, and these relationships are often further quantified in relation to biomarkers. For example, in the DSC study, one physician illustrated this point by referring to a digital programme used to determine eligibility for a particular kind of surgery. Patients were asked to input information about their weight and activity over the course of months into an app, in order to inform the decision taken with doctors on whether or not they would be a good candidate for the surgery. In the RWD study, it was pointed out by interviewees that routine health data collected for administrative purposes (e.g. ICD-10 disease classification codes used documented for reimbursement purposes) can help to identify (classify) suitable subjects for clinical studies. In the Covid-19 context, data is used to quantify viral load and classify SARS-CoV-2 infection status. In a research context, several thousands of Covid-19 patients logged their symptoms daily in an app (Sudre et al. 2020) with the goal of enabling the discovery of associations between symptoms that were previously not linked to Covid-19. Similarly, data mining exercises have been able to detect associations between comorbidities that have led to a reassessment of patient risk (Bhavnani et al. 2020; Kim et al. 2020) or an investigation of the underlying causal pathways (Lim et al. 2018). This, in turn, could change the classification of some diseases, and also shed new light on quantitative distributions.

### C: Making knowledge: generating and testing hypotheses

Data can help to test or develop hypotheses. Most forms of research (scientific, market, etc.) are hypothesis driven. Datasets suited to the research endeavor help to test hypotheses, enabling researchers to meaningfully answer a research question. But data can also generate hypotheses, e.g. in the context of inductive data mining. As the RWD study showed, real-world data from administrative healthcare processes or from publicly held disease registries can help to develop hypotheses on disease pathways or comorbidities. As the data quality in these registers is often limited (e.g. because of a lack of resources for data cleaning) or affected by bias (e.g. because data are collected for accounting, not for medical documentation), the use of these data for hypothesis testing is more challenging. Despite the excitement about RWD, the experts interviewed considered that hypothesis testing requires particular efforts in terms of data quality and will continue to rely heavily on controlled experimental environments.

The Covid-19 pandemic has led to a great number of published studies that use different kinds of health data to confirm hypotheses about infectiousness and other epidemiological parameters, disease progression, risk factors, infection fatality rate, long-term effects, and more. One study correlated US county-level Covid-19 death rates with a range of socio-economic variables, county-level health variables, modes of commuting, and climate and pollution patterns (Knittel and Ozaltun 2020, p. 5). A rather surprising finding reported in their paper is that “counties with higher home values have higher death-rates” (Baskin 2020; El-Sayed and Prainsack, 2021). This could form a hypothesis that could be investigated with other data. For example, the German Cancer Society’s Infinity idea (Rachel et al. 2021; Deutsche Krebsgesellschaft 2019) shows what a process of hypothesis generation and testing might look like. Oncological knowledge oriented to the needs of patients is created when findings from care generate new research ideas and hypotheses. These are then tested, evaluated, and published in clinical research, flow back into care, and are reevaluated in the light of the care practices.

### D: Enabling automation

In some instances, health data enables processes that were previously done by humans to be done by machines. This can eliminate the need to complete particular tasks manually, ideally allowing health professionals to spend time in other ways (even if experience teaches us that the time freed up by automating human work is often quickly absorbed by other administrative or other mandatory tasks, rather than freeing time for healthcare workers to spend with patients, learn, or rest). Data sets can be automatically updated, for example, to include the most recent pharmacological information on drug interactions or available medications, as well as for different forms of cross-referencing. It allows individual providers to contemplate clinical questions for the patient they are treating in relation to broad sets of population level data, such that providers can access and base decisions on more data than would be possible if it had to be reviewed or computed manually.

Another example are imaging datasets that can be used to “train” algorithms to quantify measurements in images or populate key findings in a report. There is a growing literature on medical image analysis algorithms automating the clustering and classification necessary for diagnostics (Anwar et al. 2018; Ker et al. 2018; Shen et al. 2017). In some cases, automation can help reduce the cost of mobilising particular forms of data for clinical use, eliminate individual cases of human error, or help to make clinical outcomes more predictable. For example, in the DSC study, one participant explained that the use of an app for migraine symptoms resulted in a much more streamlined process of patients reporting when they took medications at the onset of symptoms. This, the doctor said, was much better compared to analogue modes of logging which patients often left at home or did not have with them when symptoms began. In the Covid-19 context, we have seen data-driven automation in triage algorithms and symptom testing, contact tracing as well as the use of machine learning approaches to image analysis (e.g. of lung CT scans; see Lai et al. 2020).

### E: Changing or stabilising hierarchies and power positions

Data can change the distribution of power, agency, and resources between actors. Traditionally, health care providers have been in a position of power relative to their patients. This power is related to forms of expertise, institutional membership, and more, but also to their ability to interpret data in arriving at a diagnosis or course of action. As one physician in the DSC study noted, doctors have long been viewed as “Gods in White” in Germany, whose expertise was largely beyond the reproach of patients (DSC Interview 4). One way in which such hierarchies are shifting is with regard to healthcare data, which historically was strictly within the realm of the professional. A power shift to the patient was also indicated by an interviewee in the RWD case study. The interviewee argued that patients will be empowered in their relationship with the “Gods in White” if they know more about the performance of the medication they are prescribed (Interview A).

Increasingly, digital tools have enabled patients to both generate their own healthcare data, and to review existing healthcare data independent of their physician. Data can be mobilised by individuals or groups who have been marginalised, underrepresented, or who do not have ‘expert’ status in order to shed light on new medical conditions, gaps in therapy, and more. While such instances can increase the knowledge and agency of patients (leading to possible empowerment), there are other instances where data disempowers patients—for example when they are faced with data that may or may not say something important about their health but that they cannot interpret themselves (and may need to pay for help in interpreting). For example, one geneticist in the DSC study brought up the case of non-invasive prenatal testing (NIPT). In Germany, the companies who conduct NIPT will only do so upon request by a physician. The results are then returned to the physician, not the patient, and are generally not covered by insurance. Thus, technologies yield new forms of data and possible insight, but they also can stabilise existing hierarchies between patient and provider at the same time.

Importantly, data can also change the distribution of power, agency and resources between actors through data biases, for example, because of a quantitative misrepresentation of certain patient groups in datasets, or because of a qualitative misrepresentation (e.g. mislabeling of images). Examples abound of technological tools that were developed on the basis of biased datasets that led to worse outcomes for specific groups, often minorities (Pot et al. 2021). Data biases can also reflect human biases, such as sexist or racial stereotypes: For example, women, or people of color, are less likely to receive a diagnosis because their pain is dismissed as a cultural or gender-specific expression despite showing the same symptoms as white men. If this data is used to develop algorithms or otherwise inform clinical decisions, it will carry the same bias. Such biases can thus exacerbate existing hierarchies or inequalities.

The Covid-19 pandemic has provided other examples of how health data can affect hierarchies and power positions: epidemiological data e.g. on the infections at local level or on clusters have become part of political power play between government actors at various levels. Data on vaccine production and exports have become geopolitically important. Access to study subjects with active or past Covid-19 infections became important assets for industries active in drug and vaccine development. At a micro-level, data on infection, but also contact networks has huge effects on the lives of citizens being affected by quarantine orders.

### F: Changing or stabilising practices and procedures of people and organisations

Data has the ability to provide new insights, and to incentivise certain practices. New digital tools, such as activity trackers or other devices and smartphone applications, enable people to track patterns such as irregular heart rates, sleep, calories, activity, fertility, and more. These data are not only representing someone’s body and behavior, but they can also intervene in it. This is the case, for example, if somebody walks more (or less) because she is guided by a particular quantitative score (e.g. step count) that she wants to achieve. Moreover, the availability of this data to patients outside of clinical settings can change or stabilise individual and group practices. When applied in group settings, such as by companies wishing to incentivise their employees to get more exercise, this data can also be used to reward, or exclude, individual members of the group.

For example, in the DSC study, a physician listed examples of present and future innovation in the medical realm, ranging from avatars who conduct hospital intake or patient identity checks in the operating room, to apps to organise the arrival of ambulances in regional hospitals. They went on to note that one benefit of these innovations is that they “disrupt the system” and can help to reorganise taken-for-granted ways of doing things in healthcare. Responses from the RWD case study suggest that the increasing use of real-world data in clinical studies could alter the way data is collected in healthcare organisations. The pandemic response work in the Covid-19 context has changed how public health service is provided. Entire new components of the public health service system have been established around health data focused on contact tracing and quarantine enforcement (e.g., Mokbel et al. 2020). New data flows have been established around access systems to public and private services, and new actors have become involved in monitoring individual mobility (e.g., Buchanan et al. 2020).

### G: Proxying

Health data collection is a costly process. It requires human and machine resources including an infrastructure for observation, data documentation, and accountability. As health data is personal and sensitive (generally speaking, as well as particularly within the GDPR in the EU), it is generated in controlled environments with ethical and legal frameworks in place, e.g. regarding consent for primary or secondary use. In clinical research, for instance, an established practice of data collection is through clinical trials, very often in the form of randomised controlled trials (RCTs). There are occasions, however, where RCTs are not possible, e.g., due to lack of resources. Sometimes, an RCT might be unethical, or it might be impossible to find enough study participants. In these cases, using data from outside RCTs might serve as a proxy. One example would be the construction of a historical or so-called virtual control group out of routinely collected health data (administrative data or medical record data). This is an example of proxying. For example, in the RWD study, a respondent noted that as personalised medicine becomes more advanced, resulting in ever smaller case groups, eventually it will not be possible to complete an RCT because there are not enough patients with a particular subgroup of, for example, lung cancer (Interview C). Other examples in this vein include in silico medicine, systems medicine, virtual datasets, and projects creating data doubles for sharing, such as the Human Brain Project (https://www.humanbrainproject.eu/en/) or in education such as the Visible Human Project (Waldby 1997).

Other types of health data might also become valuable as a proxy: e.g. where digital avatars are used instead of actual study participants. These avatars or personas have similar characteristics to the actual individuals in a relevant group (i.e. measures of central tendency or variability in relevant variables are similar). They can then be used to study the population where using actual individual-level data would be impossible for ethical or legal reasons. One might also use these avatars to run simulated trials instead of actual trials. One type of health data, thus, might act as a proxy for another type of less accessible health data (Corral-Acero et al. 2020; Lehrach 2015).

Another example would be of a ‘digital twin’, such as in the case of Covid-19 (Munich School of BioEngineering, TUM 2020; see also (Braun 2021). Clinical experience during the Covid-19 pandemic shows that mechanical ventilation can save lives but also harms the patient. The basic problem is that measurements cannot be made deep in the lungs of sick patients, where ventilator-induced damage occurs. Doctors use their experience to adjust the ventilator optimally for the patient, since there is no measured value for this. Scientists at the Technical University of Munich created an anatomically-functional digital twin of the lung from individual CT scans and lung function measurements. Instead of relying purely on machine learning, they used a complex, model-based approach that is backed not only by CT images of the lungs but also by large amounts of physical knowledge. In this combination, diseased lungs can be realistically simulated, and proxy data used to inform improved clinical care. Agent-based simulation models also try to create digital twins of real humans and their behavior in order to model epidemiological dynamics (Cuevas 2020). In simulation models, digital twins or virtual study arms, the practice of proxying enables experiments or interventions that would otherwise not be possible.

### H: Predicting

Health data can become valuable for particular actors in that it allows them to model and anticipate possible situations and outcomes. Health data is used to anticipate the probability of particular outcomes. This is relevant at both the population and individual level. At the population level, for instance, as the institutional context of some of the authors as well as the background research in the context of the RWD study showed, routinely collected real-world health data (e.g. from medical health records or administrative data) can be used to predict healthcare demand, for instance as related to the prevalence of a specific disease at a certain point in the future. Such information is relevant to plan healthcare resources.

Another example is the realm of obstetrics and non-invasive prenatal testing. Using a combination of sonogram technologies, individual risk markers for the expectant mother, and extracts of fetal DNA from blood samples, the resulting data enables the healthcare provider to anticipate, with an increasing degree of accuracy, the likelihood of particular adverse health outcomes for the fetus. This information, in turn, can be valuable for some expectant mothers as they make decisions about the course of their pregnancy.

Another prominent recent example is the relevance of epidemiological and other health or health-related data to forecast the future course of a pandemic. Anticipation is also relevant in monitoring and forecasting of other infectious diseases like influenza. Other examples are individual-level models of disease progression or health outcome, or population-level models on the prevalence of non-communicable diseases or on healthcare spending. Efforts are under way, for instance, to use machine learning approaches to calculate individual risk scores for severe Covid-19 (Wynants et al. 2020). Anticipation is also relevant for biomedical research in the pharmaceutical industry, for instance when modeling the behavior of a candidate drug in silico to select possible candidate molecules for in vitro and in vivo testing.

### I: Increasing operational efficiency

Health data that is collected on personal devices such as smartphones or wearable sensors might enable users to self-monitor certain conditions, thus avoiding face-to-face contact with health providers. This reduces costs for patients, care providers and health systems (although the lack of human contact could also increase cost indirectly, e.g. by increasing social isolation and the psychological and physical problems emerging from that), and can be particularly beneficial during a pandemic. It also can be useful for particular populations, such as children managing a chronic disease like diabetes for whom routine doctors’ visits disrupts school attendance and increases the social burden of their health—as was noted by several participants in the DSC study. Similarly, patient-collected data, e.g. on lifestyle or health registry data, can make the process of identifying possible participants in a clinical study more efficient, reducing the cost of clinical research. The digitisation of processes in health care (hospital patient management, inter-professional communication, etc.) is another example where digital health data is perceived as valuable, because it enables a more efficient service delivery. As one interviewee noted in the RWD study, the increasing availability of data on patients is leading to more targeted and those more efficient clinical trials: “Because the more patients you have and the more knowledge you have about them, you can actually screen them before you even try to send them to a clinical trial.” Routine data from registers, but also health data collected through patient-facing apps can be helpful, such as in the targeted recruitment of patients for phase IV clinical trials (Interview A).

#### J: Generating financial profits

For particular actors, health data become valuable as a source of financial profit. One example are organisations specialising in collecting certain types of data to then sell the data or usage rights. Another example is health data constituting an asset in the valuation of a specific organisation by outside actors, regardless of whether the data has already been collected or not. For instance, a start-up might include a reference to privileged data access or to a novel type of data collection in its pitch, leading to financial profits for those involved. The issue here is, thus, not so much the demonstration of the quality of data, but the mere prospect of access to data or of the generation of a specific type of data. This hints at the category of aspirational value. In the DSC study, one doctor noted that one of their principal concerns with the increasing availability of digital tools and on-line, direct-to-consumer testing options, was that it was motivated by financial profit and not by medical benefit: “I’m personally quite skeptical of sending things off to somewhere where you pay privately. In Germany, usually the things that are medically justified are paid for by the insurance company […] So I’m very skeptical of things where you have to pay for yourself out of your own pocket,” (Interview 4).

One interviewee in the RWD study referenced a platform that provides a large number of self-reported drug reviews that are accessible at an aggregate level for free to patients and patient organisations, as well as government agencies, to provide insights into how patients respond to each of these drugs at the branded product level. “And of course, what we want to have is impact, so we want to make sure that we improve treatment outcomes [for] the benefits of the patient [as an] individual and of course to society in general. What we also want to improve is future medicines of the evidence that we create” (Interview A). The same data will be offered for purchase to insurance companies and pharmaceutical companies generating financial profit.

The Covid-19 pandemic is an example for the financial value of health data regarding treatment options (drug or vaccine candidates) and effectiveness. Being able to recruit a sufficient number of study participants (and maybe also being successful in *ex ante* procurement negotiations) is important to leverage large amounts of private sector investment. Similarly, health data and data infrastructures regarding testing and laboratory analysis has proven useful in generating financial value, e.g. for laboratories investing in digitally supported testing procedures.

#### K: Creating value through expectation

Health data, as other forms of data, has the characteristic of accruing value in an aspirational sense before data is actually collected and used. This can take place in a business environment, leading to financial profit (as seen in the prior category of generating financial profit). It can also take place in an academic environment where actors might get access to resources (research project participation, infrastructure access, etc.) due to the possibility of future data access or exploitation. As Hoeyer (2019) has shown, certain political actors also derive value from data by stating that more data is required in the future (such as to solve certain problems, or to address specific issues). In the RWD study, an example for the aspirational value of data was seen in the prospect of a well-documented oncological treatment, including investments in documentation and data quality control, which played a part in a reform of the institutional setup of cancer care in an Austrian region (Interview D). Better oncological data was expected to improve clinical practice, which led to institutional investments and organisational reform.

#### L: Stimulating debate and deliberation

Data offers new perspectives for looking at a problem, pattern, or change. In doing so, it can stimulate debate and deliberation over the state of reality, or what the appropriate course of action is. Very often, the availability of different kinds of data leads to deliberation, or reflection on what changes are necessary—whether in terms of large-scale epidemiological data or in individual clinical cases. For example, in the DSC study, one doctor described the debates that would likely ensue if more patients were to start employing at-home diagnostic testing and bringing those results to their doctors. The availability of at-home testing could provide data that providers were unsure what to do with, or were uncertain of if it were reliable, thus prompting a need for follow-up testing. The respondent went on to discuss other examples, such as direct-to-consumer genetic testing that might give a result of a polymorphism that increases the likelihood of developing a condition such as osteoporosis. The doctor pondered what kind of course of action this at-home genetic result might lead to when the data was brought into the clinic, as well as whether or not further testing requested by the doctor on the basis of the at-home finding would be reimbursed by an insurance company.

A look at global Covid-19 data reveals how health data can spark intense debates over questions such as whether lockdowns are justified, what constitutes appropriate ‘social distancing’ measures, or the trade-offs of school closures, in the face of the ongoing pandemic. In Covid-19, health data and its interpretation has been the center of public debate (Nagler et al. 2020). The type, availability, quality and sensibility of the data have all been issues at the agenda of civic discourse.

#### Data practices

In in looking closely at what health data help us to do, we were specifically attuned to data practices. In addition to giving rise to the roles of health data presented in the preceding section, our interviews and literature review yielded insights into how different practices are involved in creating value, or detracting from it (Table 2). Specifically, the work done in the context of (1) *quality control and improvement* was regularly seen as adding value emerging form data use. This included the standardising and cleaning datasets, recoding, or mitigating bias. The (2) *collection of large sets of high-quality data* from many units of observation/individuals was seen to enhance the value emerging from the use of such data. Also (3) the *facilitation of data linkage and reuse* (e.g. the standardisation of data and annotation, interoperability, legal foundation for reuse, storage), as well as (4) *improving accessibility* were seen to increase value for data users. Work done towards (5) *creating human–machine interfaces* and improving machine readability, and (6) the *contextualisation of datasets* (e.g. by adding metadata, fitting data and context of use by various forms of data manipulation, recoding, or anonymising) was also seen to positively influence the value emerging from data use, as was (7) *regulatory alignment* (e.g. fitting data and regulatory environment, ethics of data collection and use). Finally, (8) *claiming ownership of data* (legally and practically), and controlling data use, and were seen to either add value or detract from it, depending on the specificities of the situation (Who claims ownership? Is there individual or collective ownership? Who benefits? Who bears the cost?).

**Table 2:**
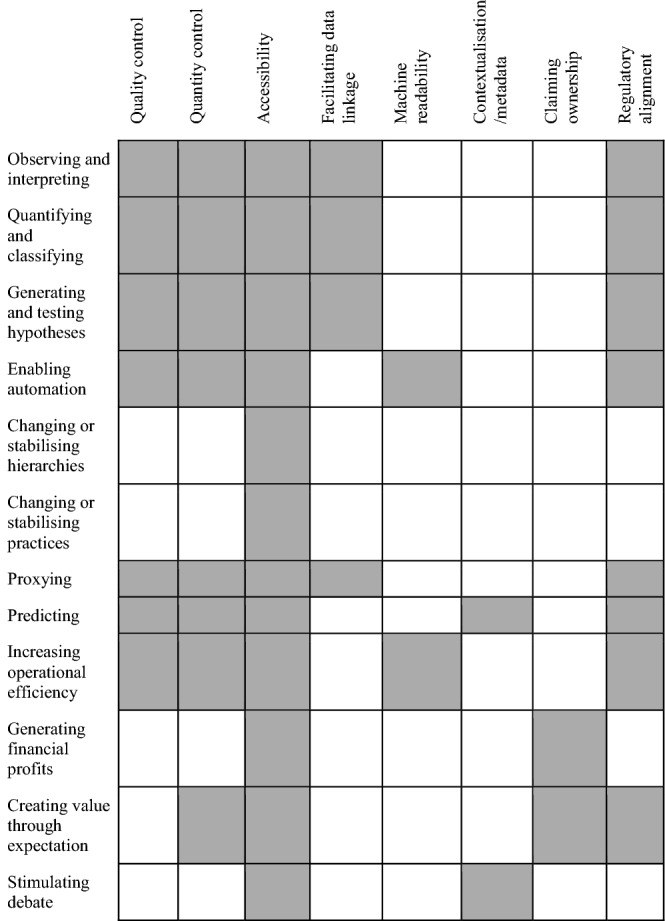
Distribution of practices affecting the value of data

Gray areas indicate how different practices (listed going across) are involved in creating value, or detracting from it across the roles of data in the typology (indicated going down)

While some of these practices apply to many of the types of data that we have identified, others are more specific. Table 2 illustrates what kinds of value-increasing practices are associated with each of the categories of data use. The associations drawn here need to be validated empirically, but our preliminary conclusion is that accessibility work (setting up data collection, etc.), regulatory alignment (ensuring a legal basis for collection and processing, etc.), quality and quantity control (sufficient data of suitable quality) are key practices when trying to increase the value of data. Others like machine readability or claiming exclusive access are more specific to certain use categories.

## Discussion and conclusions

The purpose of a specific data set is central to value. Here, our typology can be useful for better differentiating between the purpose of use: rather than speaking of ‘medical treatment’ or ‘pandemic management,’ the typology enables a more precise consideration of, say, anticipation of treatment outcome, proxy replacement of study subjects, classification of disease clusters, and more. In doing so, we argue that to assess the value of specific instances of data use, the typology provides a missing link between types of data (survey data/registry data; personal data/non-personal data; etc.) and purpose of use (pandemics management, insurance fraud detection, drug research, medical treatment, etc.). Only on this basis we can answer the question whether data use has benefits for specific groups of people, or even for society as a whole—and at what costs this may come and for whom. The proposed typology aims to assist in this work. As it is now, we anticipate that the typology will be most useful for researchers and those involved in regulation.

A change in the focus of regulation, and the categories that it uses, is necessary in our view to make regulation fit for the digital age. In an age where different datasets can be integrated and interlinked, and health-relevant inferences can be made even from health information that has no obvious link to health, we argue that it no longer makes sense for regulators to put so much weight on the characteristics of data sets (Prainsack 2017). Instead of working from a premise that certain types of data are more or less risky or sensitive, regulation should pay more attention to the practice, context, and purpose of use of datasets. As the examples illustrating the typology show, value depends on context and relationships: what value is achieved by using a specific set of health data and by whom? Aggregated sets of de-identified data that are analysed, for example, to see how healthcare services can be organised to better meet the needs of underserved populations, should be treated differently from the same datasets being used to help a commercial health insurer sort out which patients are ‘undesirable.’ In other words, a key criterion for regulation should be whether specific types of data use are in the public interest. Public interest is the case, very generally,

if it will plausibly have clear benefits for many patients, society as a whole, or future generations, and no person or group will experience significant harm. Moreover, public interest is more pronounced if the benefits are likely to materialise for underprivileged groups than for privileged people, due to the overall lower baseline and potential size of impact. (Prainsack and Buyx 2016, p. 497)

If we subscribe to the idea that regulation should pay explicit attention to whether or not a specific instance of data use is in the public interest, we need more fine-grained instruments to differentiate between different types of value that data create, and for whom. The typology proposed here should be understood as an intermediate step for assessing the public value of a specific data use. We need to better understand the roles health data assume to then link this to values and actors. This task takes on increased importance during moments such as the Covid-19 pandemic where health data has taken on new prominence in public debates over the management of health risks and inequalities, and the restriction of individual liberties in the name of public health. Knowing that the use context is classification or anticipation is not enough to decide whether a specific instance of data use is in the public interest (as anticipation can lead to better treatment choices, but also to risk-adjusted insurance fees). As the examples from the DSC study in the typology show, the use context remains a central concern for physicians working with patients using digital health data and shapes their evaluations of contingent issues of risk, harm, benefit, and more.

Focusing health data regulation primarily on types of data is not compatible with the digital age, nor does it work for public health crises such as the pandemic. In reflecting on the roles of data over the past year during the Covid-19 crisis, we have seen that different interpretations of regulation such as the GDPR have led to a patchwork response and increasing calls for data sharing in the name of public health. As Becker et al. (2020) argue, the pandemic has created a situation where there are clear reasons for attending to the public interest in health data processing. Existing provisions in the GDPR related to the processing of health data and other derogations to data subject rights on the basis of public interest could help more countries to contribute to public health research. Others have argued that the use of such exemptions may actually place an ethical obligation on policy makers and practitioners in the context of the pandemic to support collaborative research efforts in global health (McLennan et al. 2020). Focusing on the *use* and not the *type* of data (such as format or source) can help researchers, regulators, and the public to advance a more nuanced discussion of the risks, harms and benefits that emerge from the context of use. The transition to *use* turns our attention the ways that data flow, and how flows are divided and obstructed: where and how data flow, and who ends up using them, have valuation effects which are far more complicated than understandings of data as simply ‘open.’) This transition in attention helps shift focus from the legal question of whether a specific dataset can be considered anonymous (for an overview see the discussions around the GDPR; Finck and Pallas 2020; “General Data Protection Regulation [GDPR],” 2016, p. Recital 26) to the question of assessing the benefits and harms of using the dataset.

The arrival of the Covid-19 pandemic has heightened conversations around the flow of data across borders as the virus has spread, with some critics arguing that the sharing of health data is critical to a global response to the pandemic while others citing concerns around health data privacy (Cory 2020). In showing the different repertoires of valuation and types of value in health data, we believe that the typology complements scholarship on data flows literature. Yet at the same time, the typology illustrates that the flows of value and valuation do not necessarily correspond with the physical flows of data; a focus on data use instead of data type can foster an openness to the ways that data are being taken up in the world rather than a continued focus on pre-determined types within the healthcare context.

Knowing whether a certain type of routine health data is used in precision medicine to classify disease status, anticipate risk or empower patients makes a difference not only for understanding health data-related practices, but for regulating them in the light of public interest. As conversations continue around the increasing value of health data in our societies, we hope that this typology, which brings together empirical examples as well as collective reflection on the Covid-19 pandemic, can help to support a more structured reflection of thedifferent roles and values through which data use unfolds in other health contexts.
